# Rule of Law, Happiness, and Human Health: Mechanisms and Effects

**DOI:** 10.3389/fpubh.2022.908601

**Published:** 2022-07-05

**Authors:** Ling Lin, Ran Tao, Quratulain Zafar, Chaudhry Kashif Mahmood

**Affiliations:** ^1^Department of Law, School of Literature and Law, Wuchang University of Technology, Wuhan, China; ^2^Qingdao Municipal Center for Disease Control and Prevention, Qingdao, China; ^3^Department of Banking and Finance, Hasan Murad School of Management, University of Management Technology, Lahore, Pakistan; ^4^College of Business Administration, Imam Abdulrahman Bin Faisal University, Dammam, Saudi Arabia

**Keywords:** rule of law, happiness, human health, ARDL, China

## Abstract

Human health is an important concern that gradually exists in sustainable development goals. The key aim of this study is to examine the impacts of the rule of law on happiness and health using a time series data of China over the data period 1998–2020. The empirical analysis utilizes the autoregressive distributed lag (ARDL) method to find out the short and long-run effects. Findings reveal that the rule of law stimulates happiness and human health in the long-run. More internet and GDP enhance happiness and human health in the long-run. The results also showed that health expenditure and education could not boost happiness and health in the long run, but unemployment's negative effect on health. Policymakers may use our empirical results to determine applicable policies to increase human health across China provinces.

## Introduction

Happiness generally refers to pleasant and joyful emotions that people feel based on their sense of security and satisfaction, influenced by the psychological state, social environment, and external economy (1). The World Happiness Database shows that households are not as happy as expected with economic development. Since the opening and reform, the worldwide economies have perceived the accomplishments of China in economic growth (2). Meanwhile, China is also experiencing the situation of a Happiness Sluggishness predicament. The World Happiness Database reports that the national happiness of China was 5.0 during 2012, which was recorded at 7.0 during 1990. Literature reveals that the rapid increase in institutional quality has positively influenced happiness levels and human health. The scholars justify it by arguing that the level of happiness and health are strictly connected with the level of institutional development (3, 4).

The sustainable development goals committed in the United Nations mainly depend upon the political stability and institutional strengths of an individual economy (5). Institutional support in formulating and regulating health policies for controlling health security. Institutions can be reflected using different forms such as political stability, control of corruption, and the rule of law, which are the most important variables. The high quality of these indicators demonstrates a well-operative governance mechanism (6). A stable government with corruption-free norms can help formulate an influential environmental policy, and a strong rule of law can help implement it in the economy. However, if the institutional setup remains poor, firms are likely to violate environmental control protocols to maximize their profits (7).

The literature on institutions-human development nexus has focused on political stability and corruption in explaining health outcomes. However, the rule of law in explaining health outcomes is least explored. The rule of law represents the insight into how best society abides by the law, particularly in policy and regulation implementation (8). An effective rule of law ensures policy execution and bounds the firms to implement environmental laws (9). Moreover, a strong rule of the law supports the mechanism of accountability by making firms accountable for violating environmental laws.

Besides, the available studies do not provide conclusive evidence on the effect of the rule of law on human wellbeing. Analyzing the factors that determine health performance in China has become a key research agenda in recent years. A number of studies have been devoted to explaining the causes of health deterioration in China. The existing studies have focused on economic growth, health expenditure; ICT, innovation, and education (10–13). However, these studies ignored the political dimension of health problems' management. We found a small number of studies that have focused on the role of institutional quality and political economy in explaining human development (14, 15). These studies establish the links between political and institutional indicators explaining human development (16). Ongo and Song (17) confirm that governance has a positive and statically significant impact on health in African economies. Khan et al. (18) explored the impact of bank finance, institutional quality, and GDP on health and confirmed the positive effect. To our knowledge, no study has focused on the dynamic role of the rule of law in China (19). Our study's central question is whether the rule of law plays a key role in influencing human development.

The rule of law is a stronger indicator of institutional and political measures as a strong rule of the law also represents the strengths of other institutional indicators. For example, economies exhibiting a strong rule of law also demonstrate low corruption incidence (20). The rule of law has become a key challenge in BRICS economies as they are performing low on this indicator (21). According to the Majhosev (22) ranking for the rule of law, China has a score of 0.48 in 88th position, India has 0.51 in 69th position, Russia has 0.47 in 94th position, and Brazil has 0.52 in 67th position, and South Africa has 0.59 with 45th position. With the low rule of law, managing environmental goals poses serious challenges as the legislation and implication of environmental policies cannot be materialized in BRICS.

Based on the evidence above, understanding the rule of law for managing sustainable development goals in the wide spectrum is essential for designing policies. The empirical literature focusing on the rule of law as the key reason for health performance is quite limited (23). However, the present research provides fresh and novel inquiry on the association between the rule of the law and human wellbeing in China. This study contributes to the literature on institutions and environmental quality in several ways (24). First, to the best of our knowledge, this is the first empirical study exploring the role of the rule of law on happiness and health in China from 1998 to 2020. The empirical results of our research will offer new and fresh insights to numerous stakeholders in the arena of political, institutional, and environmental economics, such as political scientists, judicial experts, legal advisors, social scientists, energy economists, academic scholars, public institutes, domestic governments, regional organization, international organizations, and policymakers.

## Model and Methods

Institutional quality has multi-dimensional characteristics in each sector of the economy. Previous literature noted that institutional development increases human development by increasing happiness and human wellbeing (4, 25). Following the empirical studies of Dhrifi (4); Danish & Nawaz (26), we have constructed a basic form of happiness, and human health models are written as follows:


(1) $$\begin{array}{c} \begin{array}{l} \begin{array}{ccc} \text{Happines}{\text{s}}_{\text{t}} & = & \;\pi_{0}+\;\pi_{1}\text{RO}{\text{L}}_{\text{t}}+\pi_{2}\text{Educatio}{\text{n}}_{\text{t}}+\pi_{3}\text{Interne}{\text{t}}_{\text{t}} \end{array}\\ \begin{array}{c} \;+\pi_{4}\text{GD}{\text{P}}_{\text{t}}+\pi_{5}\text{H}{\text{E}}_{\text{t}}+\pi_{6}\text{UNEM}{\text{P}}_{\text{t}}+\varepsilon_{\text{t}} \end{array} \end{array} \end{array}$$

(2) $$\begin{array}{l} \begin{array}{ccc} \text{Healt}{\text{h}}_{\text{t}} & = & \;\pi_{0}+\;\pi_{1}\text{RO}{\text{L}}_{\text{t}}+\pi_{2}\text{Educatio}{\text{n}}_{\text{t}}+\pi_{3}\text{Interne}{\text{t}}_{\text{t}}+ \end{array}\\ \begin{array}{c} \;\pi_{4}\text{GD}{\text{P}}_{\text{t}}+\pi_{5}\text{H}{\text{E}}_{\text{t}}+\pi_{6}\text{UNEM}{\text{P}}_{\text{t}}+\varepsilon_{\text{t}} \end{array} \end{array}$$

Where happiness and health are measures of national happiness and human health that are determined by the rule of law (ROL), educational attainment (Education), Internet users (Internet), GDP per capita (GDP), health expenditure (HE), and unemployment (UNEMP). In both equations, *t* denotes period, π_0_ is the constant term, while ε_*t*_ Shows the error term. The rule of law is an important factor in explaining human development in the long run. An estimate of π_1_ should be positive. Equations (1 and 2) are a long-run model and estimates of π_1_-π_6_ reflect long-run effects of focused and control variables on happiness and human health. The short-run dynamic effects are also important. Thus, we follow Pesaran et al.'s (27) ARDL approach to estimate the long-and short-run effects in one step. Thus, we can re-express both basic equations in an error correction format, as follows:


(3) $$\begin{array}{l} \Delta {\text{Health}}_{\text{t}}=\;\pi_{0}+\;\sum_{\text{k}=1}^{\text{n}}\beta_{1\text{k}}\Delta {\text{Health}}_{\text{ t}-\text{k}}\\ +\;\sum_{\text{k=0}}^{\text{n}}\beta_{\text{2k}}\Delta {\text{Education}}_{\text{t}-\text{k}}\text{+}\sum_{\text{k=1}}^{\text{n}}\beta_{\text{3k}}\Delta {\text{Internet}}_{\text{ t}-\text{k}}\\ +\;\sum_{\text{k}=0}^{\text{n}}\beta_{\text{4k}}\Delta {\text{GDP}}_{\text{t}-\text{k}}+\sum_{\text{k}=1}^{\text{n}}\beta_{5\text{k}}\Delta {\text{HE}}_{\text{ t}-\text{k}}\\ \text{+}\sum_{\text{k}=1}^{\text{n}}\beta_{6\text{k}}\Delta {\text{UNEMP}}_{\text{ t}-\text{k}}+\;\pi_{1}{\text{Happiness}}_{\text{t}-\text{k}}+\;\pi_{2}{\text{Education}}_{\text{t}-1}\\ +\pi_{3}{\text{Internet}}_{\text{t}-1}+\;\pi_{4}{\text{GDP}}_{\text{t}-1}\text{+}\pi_{5}{\text{HE}}_{\text{t}-1}+\pi_{6}{\text{UNEMP}}_{\text{t}-1}\\ +\;\lambda .\;{\text{ECM}}_{\text{t-1}}\text{+}\varepsilon_{\text{t}} \end{array}$$

(4) $$\begin{array}{l} \Delta {\text{Health}}_{\text{t}}=\;\pi_{0}+\;\sum_{\text{k}=1}^{\text{n}}\beta_{\text{1k}}\Delta {\text{Health}}_{\text{ t}-\text{k}}\\ +\;\sum_{\text{k}=0}^{\text{n}}\beta_{2\text{k}}\Delta {\text{Education}}_{\text{t}-\text{k}}+\sum_{\text{k}=1}^{\text{n}}\beta_{3\text{k}}\Delta {\text{Internet}}_{\text{ t}-\text{k}}\\ +\;\sum_{\text{k}=0}^{\text{n}}\beta_{4\text{k}}\Delta {\text{GDP}}_{\text{t}-\text{k}}+\sum_{\text{k}=1}^{\text{n}}\beta_{\text{5k}}\Delta {\text{HE}}_{\text{ t}-\text{k}}\\ +\sum_{\text{k}=1}^{\text{n}}\beta_{6\text{k}}\Delta {\text{UNEMP}}_{\text{ t}-\text{k}}+\;\pi_{1}{\text{Health}}_{\text{t}-1}+\\ \pi_{2}{\text{Education}}_{\text{t}-1}+\pi_{3}{\text{Internet}}_{\text{t}-1}+\;\pi_{4}{\text{GDP}}_{\text{t}-1}+\pi_{5}{\text{HE}}_{\text{t}-1}\\ +\pi_{6}{\text{UNEMP}}_{\text{t}-1}+\;\lambda .\;{\text{ECM}}_{\text{t}-1}+\varepsilon_{\text{t}} \end{array}$$

Equation (3 and 4) includes short and long-run coefficient estimates, as “Δ” operator variables are reflected in short-run estimates, and long-run effects are inferred by the estimates of π_2_-π_6_ on π_1._ Pesaran et al. (27) recommend two economic tests for cointegration regarding meaningful estimates. The F-test is used for assessing the joint significance of the lagged level variables. The null hypothesis (H0 = π_1_= π_2_ = π_3_= π_4_= π_5_ = π_6_= 0) is to be verified against an alternative hypothesis (H0 ≠ π_1_≠ π_2_ ≠ π_3_≠ π_4_≠ π_5_≠ π_6_≠ 0) to determine the existence of cointegration. Besides, the *t*-test establishes the significance of λ, which must be significant and negative. Indeed, under the ARDL method, variables could be a blend of levels and first-difference. To check whether these macroeconomic variables are stationary or not, we have been applied them to DF-GLS, PP. Second, the ARDL captures the data generation process by taking a sufficient and suitable number of lags (28). This approach assumed all the variables were endogenous. Finally, the ARDL model is the most suitable approach for a small sample size, as in our empirical case.

## Data

The present study explores the transmission mechanism and impact of the rule of law on happiness and human health in the case of China. For empirical investigation, time-series data have been taken from 1998 to 2020. Definitions of variables, symbols, and descriptive statistics of variables are given in Table 1. In this study, the happiness index is adopted to measure the level of happiness. The life expectancy rate is used to measure human health. The rule of law is captured through the rule of law index. We have adopted some other determinants as control variables, for instance, education, use of the internet, GDP per capita, health expenditures, and unemployment. Average schooling years are used to measure education. As a percent of the total population, Internet users are taken to measure internet variables. GDP variable is taken in terms of constant 2015 US$. Health expenditures are taken as a percent of GDP. Unemployment is taken as a percent of the total labor force, which ILO develops. The data for analysis has been collected from the World Bank and World Happiness Report.

**Table 1:** Definitions and data description.

**Variables**	**Mean**	**Median**	**Maximum**	**Minimum**	**Std. Dev**.	**Skewness**	**Kurtosis**	**Definitions**
Happiness	4.808	4.846	5.771	4.147	0.437	0.238	2.118	Happiness index
LE	74.10	74.11	77.36	70.73	2.008	−0.057	1.821	Life expectancy at birth, total (years)
ROL	−0.451	−0.460	−0.200	−0.640	0.107	0.567	2.878	Rule of law index
Education	12.21	12.60	15.21	9.300	1.905	−0.161	1.699	Average years of schooling
Internet	2.797	3.364	4.258	0.235	1.326	−0.671	2.050	Individuals using the Internet (% of population)
GDP	8.475	8.543	9.247	7.555	0.562	−0.205	1.667	GDP per capita (constant 2015 US$)
HE	4.475	4.383	5.350	3.675	0.466	0.188	2.047	Current health expenditure (% of GDP)
UNEMP	4.336	4.520	5.000	3.240	0.480	−1.471	4.040	Unemployment, total (% of total labor force)
								(modeled ILO estimate)

## Results and Discussion

As prior testing, unit root properties of data have been confirmed by applying the DF-GLS unit root test and PP unit root test. The results outputs of both unit root tests are given in Table 2. The obtained results are similar in both unit root tests. It shows that only GDP per capita is an I (0) stationary series. However, happiness, life expectancy, the rule of law, education, internet use, health expenditures, and unemployment are I (1) stationary series. Hence, the mixture of orders of integration among data series allows us to use the ARDL approach for investigating the impact of the rule of law on human health and happiness in China. Human health and happiness are treated as dependent variables; hence, two separate ARDL models have been regressed in analysis. The results outputs of both ARDL models are given in Table 3.

**Table 2:** Unit root testing.

	**DF–GLS**			**PP**		
	**I (0)**	**I (1 )**	**Decision**	**I (0)**	**I (1)**	**Decision**
Happiness	−0.287	−3.875***	**I (** **1** **)**	0.213	−2.688*	**I (** **1** **)**
LE	1.203	−2.654**	**I (** **1** **)**	−1.254	−2.654*	**I (** **1** **)**
ROL	−1.023	−3.658***	**I (** **1** **)**	−1.023	−3.658**	**I (** **1** **)**
Education	−1.152	−3.658***	**I (** **1** **)**	−0.135	−3.654**	**I (** **1** **)**
Internet	−0.785	−2.325**	**I (** **1** **)**	−0.652	−3.788***	**I (** **1** **)**
GDP	−2.132**		**I (0)**	−2.685*		**I (0)**
HE	−0.988	−4.523***	**I (** **1** **)**	−1.102	−4.562***	**I (** **1** **)**
UNEMP	−1.036	−2.652**	**I (** **1** **)**	−2.058	−3.542**	**I (** **1** **)**

*p < 0.1; **p < 0.05; and ***p < 0.01. The bold values are decision indicators.

**Table 3:** Long and short-run estimates of happiness and health.

	**Happiness**				**Health**			
**Variable**	**Coefficient**	**S.E**	**t–Stat**	Prob.*	**Coefficient**	**S.E**	**t–Stat**	Prob.*
**Short–run**								
ROL	0.279	0.457	0.610	0.553	0.101	0.167	0.607	0.556
ROL(−1)	1.978***	0.583	3.391	0.005	0.217	0.116	1.594	0.139
Education	0.173	0.165	1.049	0.315	0.041	0.041	0.980	0.348
Internet	0.377*	0.194	1.937	0.077	0.052**	0.024	2.158	0.068
GDP	5.113**	2.163	2.364	0.036	1.075	1.478	0.727	0.482
GDP(−1)	7.697***	2.259	3.407	0.005	1.801**	0.758	2.377	0.037
HE	0.122	0.164	0.743	0.472	0.094***	0.016	5.947	0.001
HE(−1)					0.052	0.046	1.122	0.286
UNEMP	0.201	0.135	1.495	0.161	0.158	0.119	1.329	0.211
**Long–run**								
ROL	1.509***	0.307	4.922	0.000	0.138***	0.030	4.606	0.004
Education	0.115	0.103	1.119	0.285	0.229	0.307	0.747	0.471
Internet	0.252*	0.137	1.843	0.090	0.939**	0.419	2.241	0.047
GDP	1.727***	0.491	3.518	0.004	4.097**	1.592	2.573	0.026
HE	0.081	0.112	0.729	0.480	0.080	0.319	0.252	0.806
UNEMP	−0.134	0.093	1.443	0.175	−0.894**	0.388	2.303	0.042
C	8.253***	2.879	2.867	0.014	36.88***	10.57	3.487	0.005
**Diagnostics**								
F–test	7.785***				14.02***			
ECM(−1)*	−0.576*	0.294	1.957	0.071	−0.728***	0.012	60.98	0.000
LM	1.254				0.412			
RESET	0.698				0.148			
CUSUM	S				S			
CUSUM–sq	S				S			

*p < 0.1; **p < 0.05; and ***p < 0.01.

The impact of the rule of law is found to be significant and positive on happiness and human health in the long-run. These findings confirm that the rule of law positively enhances human health and happiness. A 1 percent increase in implementation of the rule of law enhances happiness level by 1.509 percent and enhances human health by 0.138 percent in the long-run. This finding is consistent with Youssef and Diab (29), who noted that quality of governance increases happiness in MENA. Finding infers that rule of law matters for a nation's happiness. This finding is consistent with Guo et al. (30), who found that environmental regulation significantly improves happiness. The rule of law positively affects happiness and human health, supported by Jain et al. (31). Findings also infer that environmental rule has simulated happiness and health by mitigating environmental pollution. A similar finding is also reported by Bonasia et al. (32) for Europe.

As displayed by statistically insignificant coefficient estimates, education reports no significant impact on happiness level and human health. The impact of the internet on happiness and human health is found positive and statistically significant. Hence, the findings confirm the contribution of ICT development to improving human health and happiness levels. It is reported that a 1 percent escalation in internet use improves happiness levels by 0.252 percent and human health by 0.939 percent in the long-run. It is found that GDP significantly and positively improves human happiness and health level in the long-run. It infers that if GDP per capita rises in China, it raises the welfare of people. That is the major reason behind the improvement is human happiness and health level. The results reveal that a 1 percent improvement in GDP per capita raises happiness level by 1.727 percent and health level by 4.097 percent in the long-run. Current health expenditures report no significant impact on happiness level and human health, as displayed by statistically insignificant coefficient estimates. Unemployment impact is found to be significant and negative on human health in the long-run. It shows that unemployment reduces people's affordability due to the unavailability of financial resources. Thus, their health suffers. The obtained results show that 1 percent intensification in unemployment brings a 0.894 percent reduction in human health. Furthermore, no significant association is found between unemployment and happiness levels in the long-run.

The rule of law reports no significant impact on happiness level and human health in the short-run, as confirmed by statistically insignificant coefficient estimates. However, the rule of law effect becomes significantly positive on happiness level after 1 year lag. Similarly, education reports no significant impact on happiness level and human health, as displayed by insignificant coefficient estimates of education in the short-run. The use of the internet contributes positively to improving happiness levels and human health in the short-run. However, GDP is found to be positively associated with happiness level in the short-run. The association of health and GDP is found insignificant in the short-run, while it becomes significantly positive after 1 year lag. Health expenditures bring significantly positive improvement in human health in the short-run. But the association between happiness and health expenditures is found to be statistically insignificant in the short-run. Unemployment reports no significant impact on happiness level and human health, as confirmed by statistically insignificant coefficient estimates of unemployment in the short-run.

The lower panel of Table 2 delivers the output of diagnostics tests. The application of these tests is mandatory to validate the results of the ARDL approach. The long-term cointegration is confirmed in both models, as displayed by the F-test and ECM test findings. Moreover, the negative sign attached to ECM tests results confirmed the convergence tendency toward equilibrium in case of any divergence. No auto-correlation problem appeared in models, as confirmed by the output of the LM test. Additionally, models are correctly specified, and stability condition is also fulfilled as described by the results of the Ramsey RESET test and CUSUM and CUSUM-sq tests. The results from the causal analysis are described in Table 4. The results also show bidirectional causal nexus between ROL and health, ROL and happiness.

**Table 4:** Results of causality test in China.

**Null hypothesis:**	**F-Stat**	**Prob**.	**Null hypothesis:**	**F-Stat**	**Prob**.
ROL → LE	4.997	0.021	ROL → HAPPINESS	8.155	0.004
LE → ROL	2.847	0.088	HAPPINESS → ROL	4.461	0.029
EDUCATION → LE	3.779	0.045	EDUCATION → HAPPINESS	7.002	0.007
LE → EDUCATION	3.682	0.048	HAPPINESS → EDUCATION	1.093	0.359
INTERNET → LE	0.152	0.860	INTERNET → HAPPINESS	3.239	0.066
LE → INTERNET	0.356	0.706	HAPPINESS → INTERNET	0.021	0.979
GDP → LE	1.578	0.237	GDP → HAPPINESS	6.322	0.010
LE → GDP	1.064	0.368	HAPPINESS → GDP	4.183	0.035
HE → LE	1.083	0.362	HE → HAPPINESS	1.883	0.184
LE → HE	1.433	0.268	HAPPINESS → HE	1.607	0.231
UNEMP → LE	2.707	0.097	UNEMP → HAPPINESS	0.143	0.868
LE → UNEMP	2.444	0.119	HAPPINESS → UNEMP	1.978	0.171
EDUCATION → ROL	7.331	0.006	EDUCATION → ROL	7.331	0.006
ROL → EDUCATION	0.032	0.969	ROL → EDUCATION	0.032	0.969
INTERNET → ROL	1.465	0.260	INTERNET → ROL	1.465	0.260
ROL → INTERNET	0.300	0.745	ROL → INTERNET	0.300	0.745
GDP → ROL	3.180	0.069	GDP → ROL	3.180	0.069
ROL → GDP	0.406	0.673	ROL → GDP	0.406	0.673
HE → ROL	1.450	0.264	HE → ROL	1.450	0.264
ROL → HE	5.523	0.015	ROL → HE	5.523	0.015
UNEMP → ROL	0.242	0.788	UNEMP → ROL	0.242	0.788
ROL → UNEMP	0.415	0.668	ROL → UNEMP	0.415	0.668
INTERNET → EDUCATION	0.964	0.403	INTERNET → EDUCATION	0.964	0.403
EDUCATION → INTERNET	0.367	0.699	EDUCATION → INTERNET	0.367	0.699
GDP → EDUCATION	5.057	0.020	GDP → EDUCATION	5.057	0.020
EDUCATION → GDP	1.404	0.274	EDUCATION → GDP	1.404	0.274
HE → EDUCATION	0.322	0.729	HE → EDUCATION	0.322	0.729
EDUCATION → HE	1.136	0.346	EDUCATION → HE	1.136	0.346
UNEMP → EDUCATION	0.920	0.419	UNEMP → EDUCATION	0.920	0.419
EDUCATION → UNEMP	3.810	0.044	EDUCATION → UNEMP	3.810	0.044
GDP → INTERNET	3.384	0.060	GDP → INTERNET	3.384	0.060
INTERNET → GDP	2.407	0.122	INTERNET → GDP	2.407	0.122
HE → INTERNET	0.250	0.782	HE → INTERNET	0.250	0.782
INTERNET → HE	0.488	0.623	INTERNET → HE	0.488	0.623
UNEMP → INTERNET	0.107	0.899	UNEMP → INTERNET	0.107	0.899
INTERNET → UNEMP	2.378	0.125	INTERNET → UNEMP	2.378	0.125
HE → GDP	0.005	0.995	HE → GDP	0.005	0.995
GDP → HE	20.74	0.000	GDP → HE	20.74	0.000
UNEMP → GDP	6.410	0.009	UNEMP → GDP	6.410	0.009
GDP → UNEMP	3.901	0.042	GDP → UNEMP	3.901	0.042
UNEMP → HE	0.229	0.798	UNEMP → HE	0.229	0.798
HE → UNEMP	0.670	0.526	HE → UNEMP	0.670	0.526

*p < 0.1; **p < 0.05; and ***p < 0.01.

## Conclusion and Implications

Human health and happiness are important concerns gradually rising among researchers and policy developers. Good health and happiness contribute significantly to the process of development. The literature on the association between human health and economic development has increased in a few decades; however, no attempt has been made to assess the role of the rule of law in determining human health and happiness levels. However, institutional quality impact on health outcomes is explored, but the happiness concern is still neglected. Hence, the main aim of this research is to fill this gap by investigating the impact of the rule of law on human health and happiness in case of China. Using the ARDL approach for time series data ranging from 1998 to 2020, the findings reveal that the rule of law positively raises happiness levels and human health in the long-run. Hence, it is concluded that proper implementation of the rule of law in every segment of the economy can significantly improve the wellbeing of the public that improves their level of happiness and health. However, no significant association is found between the rule of law, happiness, and health in the short-run. The study has incorporated some important determinants as control variables. The findings report that the internet and GDP improve the level of health and happiness in the long-run. However, unemployment reduces the level of health in the long-run. Health expenditure and education report an insignificant impact on human health and happiness in the long-run. Internet and GDP bring a significant increase in human health in the short-run. However, the internet and current health expenditures increased the level of health in the short-run.

The study provides the following implications based on these findings. A good institution is an important channel that affects China's human health status. Moreover, improving the quality of an institution is not only significant for human health, but is also crucial for national happiness. China must improve governance indicators such as law and order and fight corruption to upsurge human health significantly. Thus, it is essential to increase the implementation of environmental law to improve human health. A strong economic system is vital for the execution of institutional policies. The Chinese government should strengthen the institutional framework to increase human development on a priority basis.

There are also some limitations in our empirical research. This study is only limiting empirical analysis for China. Future studies should also cover other Chinese provinces and nations to examine the nexus. This study concentrated only on one institutional factor, in the upcoming we intend to focus on each institutional factor. Upcoming studies can scrutinize the nexus between institutional factors and human wellbeing.

## Data Availability Statement

Publicly available datasets were analyzed in this study. This data can be found here: https://data.worldbank.org.

## Author Contributions

LL and RT: conceptualization, software, data curation, and writing—original draft preparation. QZ: methodology, writing—reviewing, and editing. CM: visualization and investigation. All authors contributed to the article and approved the submitted version.

## Conflict of Interest

The authors declare that the research was conducted in the absence of any commercial or financial relationships that could be construed as a potential conflict of interest.

## Publisher's Note

All claims expressed in this article are solely those of the authors and do not necessarily represent those of their affiliated organizations, or those of the publisher, the editors and the reviewers. Any product that may be evaluated in this article, or claim that may be made by its manufacturer, is not guaranteed or endorsed by the publisher.

## References

[1] Diener E Biswas-Diener R. Psychological empowerment and subjective well-being. Measuring empowerment: Cross-disciplinary perspectives. Washington, DC: The World Bank (2005) p. 125. 10.1037/e597202012-007

[2] Zwolinski J. Happiness around the world. Cross-Cultural Psychology: Contemporary Themes and Perspectives. Washington, DC: American Psychological Association (2019) p. 531–545. 10.1002/9781119519348.ch26

[3] Bodnar BE Claassen CW Solomon J Mayanja-Kizza H Rastegar A. The effect of a bidirectional exchange on faculty and institutional development in a global health collaboration. PLoS ONE. (2015) 10:e0119798. 10.1371/journal.pone.0119798 25799567 PMC4370667

[4] Dhrifi A. Public health expenditure and child mortality: does institutional quality matter? J Knowl Econ. (2020) 11:692–706. 10.1007/s13132-018-0567-4

[5] Kabeer N. Gender Mainstreaming in Poverty Eradication and the Millennium Development Goals: A handbook for policy-makers and other stakeholders. London: Commonwealth Secretariat Marlborough House Pall Mall (2003). 10.14217/9781848598133-en

[6] Greenidge K McIntyre MMA Yun H. Structural Reform and Growth: What Really Matters? Evidence from the Caribbean International Monetary Fund. Washington, DC: International Monetary Fund (2016). 10.2139/ssrn.2844160

[7] Tao R Su C-W Naqvi B Rizvi SKA. Can Fintech development pave the way for a transition towards low-carbon economy: a global perspective. Technol Forecast Soc Change. (2022) 174:121278. 10.1016/j.techfore.2021.121278

[8] Peerenboom R. China's long march toward rule of law. Cambridge University Press. (2002). 10.1017/CBO9780511493737

[9] McAllister L. Making Law Matter: Environmental Protection and Legal Institutions in Brazil. Stanford University Press. (2008). 10.11126/stanford/9780804758239.001.0001

[10] Filmer D Pritchett L. The impact of public spending on health: does money matter? Soc Sci Med. (1999) 49:1309–23. 10.1016/S0277-9536(99)00150-1 10509822

[11] Dutta UP Gupta H Sengupta PP. ICT and health outcome nexus in 30 selected Asian countries: Fresh evidence from panel data analysis. Technol Soc. (2019) 59:101184. 10.1016/j.techsoc.2019.101184

[12] Kouton J Bétila RR Lawin M. The impact of ICT development on health outcomes in Africa: Does economic freedom matter? J Knowl Econ. (2021) 12:1830–69. 10.1007/s13132-020-00689-3

[13] Majeed MT Khan FN. Do information and communication technologies (ICTs) contribute to health outcomes? An empirical analysis. Qual Quant. (2019) 53:183–206. 10.1007/s11135-018-0741-6

[14] Rehmat S Majeed MT Zainab A. Panel data analysis of institutional quality and population health outcomes. Empirical Economic Review. (2020) 3:21−42. Available online at: https://ojs.umt.edu.pk/index.php/eer/article/view/43531995597

[15] Ullah K Majeed MT. District-level multidimensional poverty and human development in the case of Pakistan: does institutional quality matter? Geo J. (2022) 1–21. 10.1007/s10708-022-10600-z

[16] Singh BP Yadava AK. Technical efficiency of financial inclusion and human development: Insights from the Indian states. Economic Notes. (2021) e12199. 10.1111/ecno.12199

[17] Ongo Nkoa BE Song JS. Does Institutional Quality increase inequalities in Africa? J Knowl Econ. (2021) 1–32. 10.1007/s13132-021-00771-4

[18] Khan MA Gu L Khan MA Oláh J. Natural resources and financial development: The role of institutional quality. J Multinatl Financ Manag. (2020) 56:100641. 10.1016/j.mulfin.2020.100641

[19] Su C-W Qin M Rizvi SKA Umar M. Bank competition in China: a blessing or a curse for financial system? Economic Research-Ekonomska IstraŽivanja. (2021) 34:1244–64. 10.1080/1331677X.2020.1820361

[20] Umar M Ji X Kirikkaleli D Xu Q. COP21 Roadmap: Do innovation, financial development, and transportation infrastructure matter for environmental sustainability in China? J Environ Manage. (2020) 271:111026. 10.1016/j.jenvman.2020.111026 32778306

[21] Su C-W Xie Y Shahab S Faisal CMN Hafeez M Qamri GM. Towards achieving sustainable development: role of technology innovation, technology adoption and CO2 emission for BRICS. Int J Environ Res Public Health. (2021) 18:277. 10.3390/ijerph18010277 33401479 PMC7795817

[22] Majhosev A. World Justice Project, Rule of Law Index 2021. Washington, DC: World Justice Project, NW (2021)

[23] Li C Su C-W Altuntaş M Li X. COVID-19 and stock market nexus: evidence from Shanghai stock exchange. Economic Research-Ekonomska IstraŽivanja. (2021) 0:1–14. 10.1080/1331677X.2021.1941181

[24] Chang T Chang Y-C Liu T-Y Su C-W Wang M-C. Treatment after Pollution? Ener Environ. (2021) 0958305X211053289. 10.1177/0958305X211053289

[25] Boettke P Subrick JR. Rule of law, development, and human capabilities. Supreme Court Economic Review. (2003) 10:109–26. 10.1086/scer.10.114714035112229

[26] Danish MH Nawaz SMN. Does institutional trust and governance matter for multidimensional well-being? Insights from Pakistan. World Dev Perspect. (2022) 25:100369. 10.1016/j.wdp.2021.100369

[27] Pesaran MH Shin Y Smith RJ. Bounds testing approaches to the analysis of level relationships. J Appl Economet. (2001) 16:289–326. 10.1002/jae.616

[28] Usman A Ozturk I Hassan A Zafar SM Ullah S. The effect of ICT on energy consumption and economic growth in South Asian economies: an empirical analysis. Telemat Informat. (2021) 58:101537. 10.1016/j.tele.2020.10153732524409

[29] Youssef J Diab S. Does quality of governance contribute to the heterogeneity in happiness levels across MENA countries? J Bus Socio- Econ Dev. (2021) 10.1108/JBSED-03-2021-0027

[30] Guo S Wang W Zhang M. Exploring the impact of environmental regulations on happiness: new evidence from China. Environ Sci Pollut Res. (2020) 27:19484–501. 10.1007/s11356-020-08508-7 32215800

[31] Jain M Sharma GD Mahendru M. Can I sustain my happiness? A review, critique and research agenda for economics of happiness. Sustainability. (2019) 11:6375. 10.3390/su11226375

[32] Bonasia M De Simone E D'Uva M Napolitano O. Environmental protection and happiness: a long-run relationship in Europe. Environ Impact Assess Rev. (2022) 93:106704. 10.1016/j.eiar.2021.106704

